# Single-cell RNA-sequencing in asthma research

**DOI:** 10.3389/fimmu.2022.988573

**Published:** 2022-11-29

**Authors:** Weifeng Tang, Mihui Li, Fangzhou Teng, Jie Cui, Jingcheng Dong, Wenqian Wang

**Affiliations:** ^1^ Department of Integrative Medicine, Huashan Hospital, Fudan University, Shanghai, China; ^2^ The Institutes of Integrative Medicine, Fudan University, Shanghai, China

**Keywords:** asthma, scRNA-seq, mechanism, immune cell, structural cell

## Abstract

Asthma is a complex and heterogeneous disease with multicellular involvement, and knowledge gaps remain in our understanding of the pathogenesis of asthma. Efforts are still being made to investigate the immune pathogenesis of asthma in order to identify possible targets for prevention. Single cell RNA sequencing (scRNA-seq) technology is a useful tool for exploring heterogeneous diseases, identifying rare cell types and distinct cell subsets, enabling elucidation of key processes of cell differentiation, and understanding regulatory gene networks that predict immune function. In this article, we provide an overview of the importance of scRNA-seq for asthma research, followed by an in-depth discussion of the results in recent years, in order to provide new ideas for the pathogenesis, drug development and treatment of asthma.

## Introduction: Current status of asthma treatment

Asthma is a heterogeneous disease characterized by chronic airway inflammation. Multiple types of cells and their products play an important role in its pathogenesis (1). Asthma is a major public health challenge, affecting approximately 300 million people worldwide, and this number is constantly rising (2, 3). In addition to high mortality, asthma also reduces the quality of life and physical functioning of asthmatics and imposes an economic burden (4). The combination of beta2-adrenergic receptor agonists and steroids is effective in the treatment of asthma, but some patients have poor response or tolerance to beta2-agonists and glucocorticoids due to disease heterogeneity or individual differences (5). Corticosteroids also do not cure or significantly alter the course of asthma, as asthma symptoms return quickly after the drug is discontinued (6). Systemic corticosteroid use may also cause hypothalamic-pituitary-adrenal axis dysfunction. By contrast, treating asthma with biological agents may increase costs and side effects (7–9). Therefore, the pathogenesis of asthma needs to be further studied. The emergence of scRNA-seq technology provides a new research idea and solution for the comprehensive and systematic study of effector cells and key molecular mechanisms of asthma.

## The complex pathogenesis of asthma

The pathogenesis of asthma is partially unknown, and genome-wide association studies have shown that immune responses and epithelial barrier dysfunction contribute to the pathogenesis of asthma (10, 11).

The pathological process of asthma is involved in a variety of cells and cellular components, including dendritic cells (DCs), mast cells, eosinophils, T lymphocytes, B lymphocytes and neutrophils, as well as epithelial cells, which interact with inflammatory cells and the external environment, leading to chronic airway inflammation, reversible airflow limitation, airway hyperresponsiveness, and airway remodeling. When exposed to allergens, epithelial cells monitor specific antigens and release pro-inflammatory factors that promote the recruitment and activation of DCs, thereby inducing allergen-specific type 2 T helper (Th2) cell activation (12). Upon re-exposure to allergens, Th2 effector cells are stimulated and activated to secrete type 2 inflammatory factors such as IL-4, IL-5 and IL-13, recruit and activate eosinophils and mast cells, and stimulate B lymphocytes to produce IgE, which mediates eosinophil inflammation and regulates cellular and humoral immunity (13, 14). Type 2 innate lymphoid cells (ILC2s) are also important mediators of asthma by producing IL-5, IL-13 and cross­regulating conventional T cells (15, 16). Increased neutrophils and enhanced Th17 immune responses are also considered to be involved in the pathological progression of severe asthma (17–19).

Airway epithelial cells are located at the junction between the inhalation environment and the lung. They are the first barrier to protect the human body from pathogens, and are crucial for the occurrence and development of asthmatic airway inflammation (20). When the epithelial barrier is disrupted, it promotes the secretion of growth factors from epithelial cells, which activates fibroblasts and myofibroblasts, which in turn promotes excessive collagen deposition, leading to increased smooth muscle mass (21). In addition, epithelial cells can produce cytokines crucial for innate immunity and promote immune cell activity. For example, IL-25 and IL-33 secreted by airway epithelium can activate immune cells and recruit inflammatory cells to initiate Th2-type cell responses or ILC2 activation. On the other hand, IL-13, a type 2 inflammatory factor that plays an important role in structural cells (22, 23), such as epithelial goblet cell metaplasia (24) and mucin glycoprotein MUC5AC secretion (25, 26), is a critical factor in lethal airway obstruction (25, 27).

In conclusion, the pathological process of asthma is participated by a variety of cells and their components, such as eosinophils, T lymphocytes, B lymphocytes, macrophages, neutrophils and epithelial cells, which interact with the external environment, leading to chronic airway inflammation, reversible airflow limitation, and airway remodeling in asthmatic patients, showing the diversity of asthma phenotypes and the complexity of pathogenesis.

## Application and research progress of scRNA-seq technology in asthma pathogenesis

Transcriptome sequencing is widely used to study the mechanisms of asthma. Traditional transcriptome sequencing methods based on blood or tissue samples require a large number of cell populations, presenting the average expression levels in all samples, and cannot present the characteristics of individual cells. Therefore, the pathogenesis of heterogeneous disease has not been fully elucidated. ScRNA-seq is a technology that parses RNA sequences at the single-cell level. It takes a single cell as a unit, separates the cell population in a tissue or body fluid sample into a single cell, performs whole transcriptome amplification and high-throughput sequencing, and obtains the corresponding data and information analysis. This disruptive technology enables higher-resolution analysis of cellular differences and a better understanding of how individual cells function in their specific microenvironment, specific therapeutic and disease context. In addition, scRNA-seq predicts cell-to-cell interactions and state changes in extraordinary detail and characterizes the molecular cellular phenotypes (or cell “states”) (20). Conceivably, scRNA-seq technique can achieve more unique targets than traditional approaches, such as identifying rare cells, defining disease subtypes, discovering new cellular markers, characterizing cellular heterogeneity and subsets, elucidating disease mechanisms, and enabling precise and personalized drugs.

**Figure 1:**
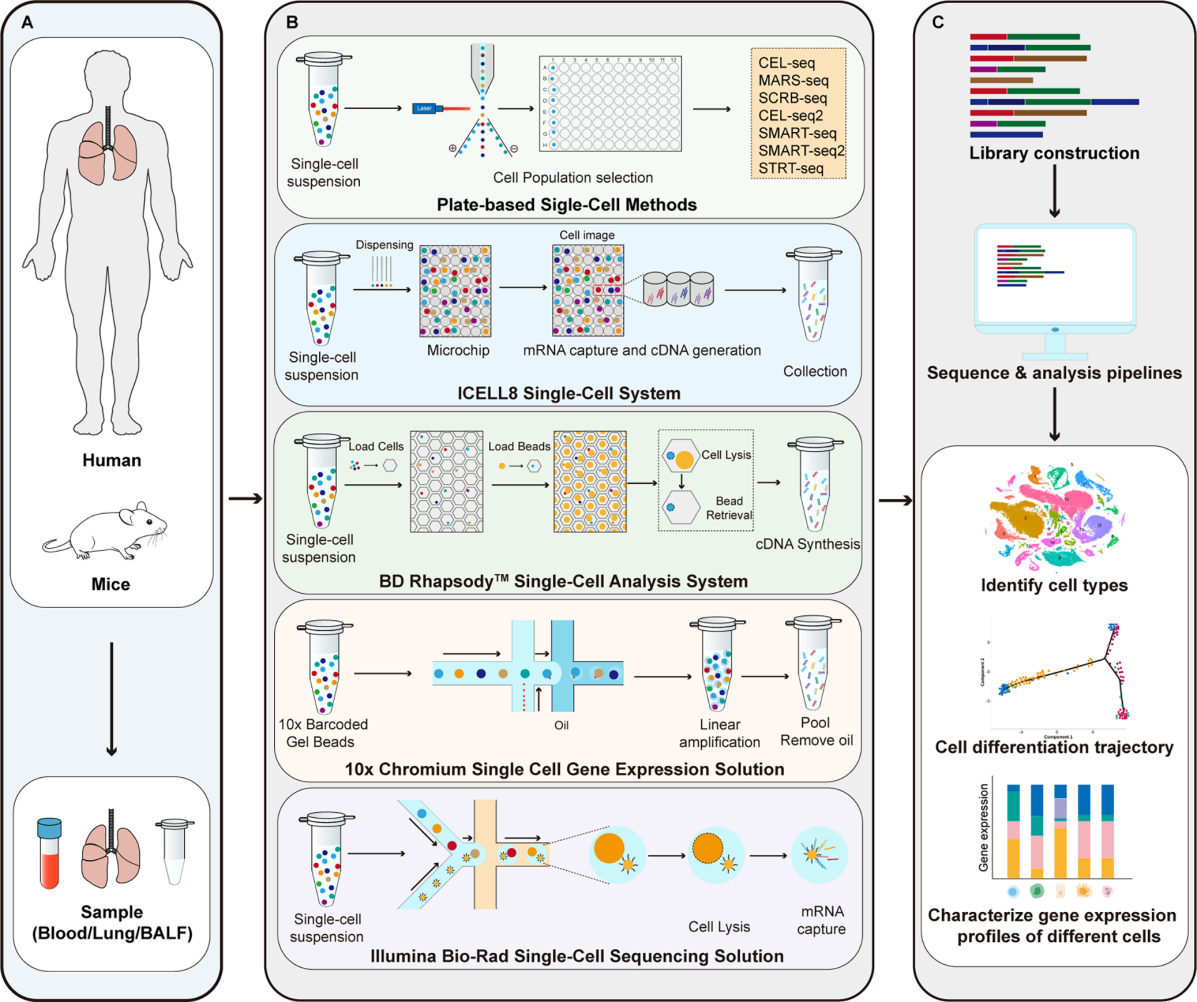
Principle of scRNA-seq technologies. **(A)** Sample preparation; **(B)** Single-cell library construction and sequencing; **(C)** Bioinformatics data analysis. CEL-seq, cell expression by linear amplification and sequencing; MARS-seq, massively parallel single-cell RNA sequencing; SCRB-seq, single-cell RNA barcoding and sequencing; SMART-seq, switching mechanism at 5’ end of the RNA transcript; STRT-seq, single-cell tagged reverse transcription sequencing.

As shown in **Figure 1**, the main steps of scRNA-seq include single cell preparation, single-cell library construction and sequencing, and bioinformatics data analysis (28). There are a variety of single-cell isolation techniques. Nucleic acid is obtained after isolation and lysis of single-cell and then amplified for subsequent sequencing. Various single cell isolation methods have been developed depending on the state of the sample, the number of cells required, and the purpose of the analysis (**Table 1**). Based on microdroplet or microfluidic chip technology, many manufacturers have launched their own high-throughput scRNA Seq platforms. At present, there are four mainstream commercial platforms: ICELL8 Single-Cell System, BD Rhapsody™ Single-Cell Analysis System, Illumina Bio-Rad Single-Cell Sequencing Solution and 10x Chromium Single Cell Gene Expression Solution.

**Table 1:** List of single cell preparation methods.

	Method for single cell separation		Advantage		Shortage	
Low throughput	Limiting dilution (29)		Simple operation; Good repeatability; Low cost		Untargeted screening results in isolation errors or cell loss	
	Micromanipulator (30)		Simple operation; Low cost; Suitable for suspension cells		Isolation errors or mechanical cell damage	
	Laser capture microdissection (31)		Maintain cell morphological structure and keep spatial location information		Cell integrity damage	
High throughput	Fluorescence-activated cell sorting (32) (FACS)		The application field is wide; High sensitivity; Can screen rare cells or small cells		Large sample dosage; Lost part of cell function and intercellular interaction and other information; Low intensity fluorescent samples could not be detected;	
	Magnetic-activated cell sorting (33) (MACS)		The application field is wide; Time saving; High sensitivity; Can screen rare cells or small cells		Lost part of cell function and intercellular interaction and other information;	
	Microfluidics control (34)		High throughput; Flexible operation; Small sample demand		High cost	

**Figure 2:**
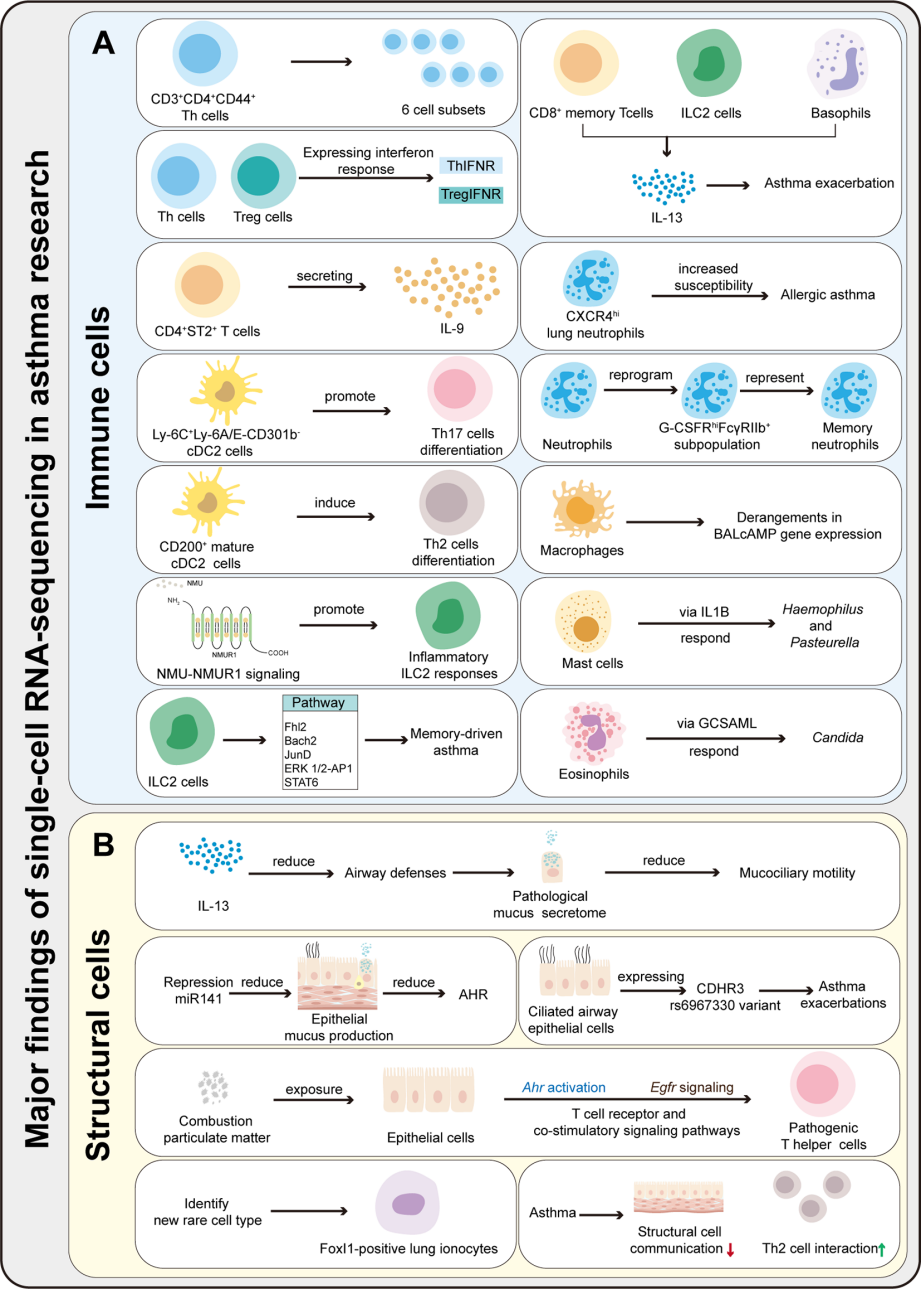
Summary of the major findings resulting from scRNA-seq application in asthma research. **(A)** ScRNA-seq can be used to characterize the heterogeneity and function of immune cells, identify rare cells and new cell subsets, and explores immune cells differentiation mechanisms and pathways in asthma. **(B)** ScRNA-seq can be used to characterize the function of structural cells, identify rare cells, and explore the communication between immune and structural cells.

ScRNA-seq can obtain the transcriptome expression data of each cell, accurately present the differences in gene expression between different cells, so as to re-understand the role of various tissues and organs in the pathogenesis of disease at the single-cell level, and has irreplaceable advantages in the study of heterogeneous diseases (35). ScRNA-seq has been applied in the study of asthma, mainly targeting immune cells (T cells, DCs, ILCs, macrophages, mast cells, neutrophils, eosinophils, etc.) and affected histiocytes (airway epithelial cells, goblet cells, etc.) involved in the pathogenesis. Therefore, it provides new technical support for elucidating its pathogenesis and exploring new therapeutic targets. This article categorizes published studies by cell type to reveal the role of different cell populations and interactions between populations in asthma pathogenesis (**Table 2**). The main findings of scRNA-seq in asthma are summarized in **Figure 2**. The key words used in the literature retrieval were “asthma” and “single cell transcriptome” or “scRNA”. We manually screened the literatures, excluding reviews and literature with non-asthmatic patients or non-asthmatic models.

**Table 2:** Summary of scRNA-seq in asthma.

Cell classification	References	Species	Tissue	Cell Type	Molecules/pathways identified	Features of the study	Cell isolation	Transcriptomic method/platform	ScRNA-seq data source ID
Immune cells	2020 (36), Grégory Seumois	Human	PBMCs	T cell	*TNFSF10*, IL-9	IL9-expressing Th2, ThIFNR, TregIFNR subsets were revealed in asthma	MACS, FACS	10x Genomics, Illumina HiSeq 2500	GSE146170
Immune cells	2021 (37), Ailu Chen	Human	PBMCs	CD4^+^ T cells, CD8^+^ T cells, NK cells, B cells, DCs, monocytes	JAK1	CD4^+^ T cells were the main cell type in severe asthma and demonstrated a pro-inflammatory profile characterized by increased JAK1 expression	Lymphoprep and SepMate™-15(IVD)	Drop-Seq, Illumina Hi-Seq 2000	GSE172495
Immune cells	2020 (38), Daniel Spakowicz	Human	Sputum	Mast cells, eosinophils	–	Mast cells respond to *Haemophilus* and *Pasteurella via* IL1B and that eosinophils respond to *Candida via* GCSAML	Hypertonic saline, Fluidigm C1 medium-sized channel	Illumina HiSeq 2000	dbGaP PRJNA611097
Immune cells	2021 (39), Hui Li	Human	BALF	CD8^+^ T cells, monocyte, macrophage	Eukaryotic initiation factor 2, ephrin receptor, C-X-C chemokine receptor type 4 signaling	Identified severe asthma–associated genes that are differentially expressed by multiple cell clusters	FACS	BD Rhapsody Single-Cell Analysis System	–
Immune cells	2017 (40), Antonia Wallrapp	C57Bl/6J mice	Lung	ILC2s	*Nmur1*, NMU–NMUR1 signaling	Promotes inflammatory ILC2 responses	FACS; MACS	10× Genomics; SMART-Seq2	GSE102299
Immune cells	2019 (41), Coraline Radermecker	BALB/c mice	Lung	CXCR4^hi^ lung neutrophils	–	Increased susceptibility to allergic asthma	FACS; MACS	10× Genomics	ArrayExpress database E-MTAB-6902
Immune cells	2021 (42), Mukesh Verma	C57BL/6 mice	Lung	Macrophages, NK cells, DCs, endothelial cells, ILC2s	*Nr4a2, Zeb1, Bach2, JunD, Fhl2, FosB, Stat6, Srebf2, MPP7*	ILC2s are essential in memory-driven asthma	FACS	ICELL8 platform, Illumina HiSeq 4000	GSE172258
Immune cells	2021 (43), Lingli Wang	BALB/c mice	Lung	CD8^+^ memory T cells, ILC2, basophils	STK11, NFE2L2, NF-κB(NF-κB1, NF-κB2, Rela, Relb, Rel),EIF2, PKC-, Ephrin receptor-, phospholipase C-, Rho-, Rac-,Tec kinase, fMLP signaling	IL-13 produced by CD8^+^ memory T cells, ILC2, and basophils is a key cytokine in driving the pathogenesis of asthma exacerbation	FACS	10x Genomics, Illumina HiSeq 4000	-
Immune cells	2021 (44), Kaveh Moghbeli	Human	Lung	Alveolar macrophages, monocytes, interstitial macrophage, NK cell	*CREM, CD83, BTG3, NAMPT, RASGEF1, AREG, HBEGF, EREG, VEGF, CXCL2, REL*, cAMP signaling	Prolonged β-agonist exposure is associated with decreased expression of genes involved in cAMP signaling	–	10x Genomics	GSE136587
Immune cells	2022 (45), Benjamin J. Ulrich	C57BL/6 mice	Lung	Th subsets, ILC2s	*Il9r, Il1rl1, Scgb1a1, Mgp, Retnla, Scgb1a1*	IL-9-secreting CD4^+^ ST2^+^ T cells were defined as producing multiple type 2 cytokines that are distinct from Th2 cells	MACS, FACS	10x Genomics, NovaSeq 6000	GSE190795
Immune cells	2022 (46), Zhiwei Li	C57BL/6J mice	Lung	Neutrophils	Il4ra, Runx1 and Cebpb, Jak2, Ctnnb1, Tet2, Toll-like receptor signaling, cAMPTNF response, TNF response, JAK- STAT cascade	Asthma may lead to an expansion of the G-CSFR^hi^FcγRIIb^+^ subset of memory neutrophils	Enzymolysis approach	10x Genomics, NovaSeq 6000	CRA004586
Immune cells	2020 (47), Jian Jiang	Human	Lung parenchyma	Mast cells	*TPSB2, TPSAB1, PTGS2, HPGDS, CPA3*	Mast cells were increased in the asthma patients and reduced in asthma patients with ICS treatment	–	Illumina HiSeq 4000	European Genome-phenome Archive EGAS00001002649
Immune cells	2021 (48), Gentaro Izumi	C57BL/6J mice	Lung, bone marrow	DCs, preDCs, T cells	*Ly-6C^+^, Ccr7, Il1b*	Th17 and Th2 differentiation are promoted by lung cDC2 at distinct stages of maturation	AutoMACS, FACS, single-cell ChIP	10x Genomics	GSE156527
Immune cells	2019 (49), Christopher Andrew Tibbitt	C57BL/6J mice	Mediastinal lymph node, lung, airways	CD3^+^CD4^+^CD44^+^Th	*Cd200r1, Il6, Plac8, Igfbp7*	Glycolysis and lipid metabolism in driving Th2-cell-mediated immune responses	FACS	SMART-Seq2	GSE131935
Structural cells	2021 (50), Sana Siddiqui	Human	Airway epithelial	Primary HBECs, airway epithelial	Goblet cell genes, IL-13 signaling	Repression miR141 downregulates epithelial mucus production *in vivo* and *in vitro* and reduces airway hyperresponsiveness	Rotating shaker, 100 μM filter	10x Genomics	GSE164015
Structural cells	2018 (51), Daniel T. Montoro	C57BL/6J mice	trachea	Airway epithelial cells, FoxI1-positive pulmonary ionocyte	*Foxi1,Cftr*	Disrupt airway fluid and mucus physiology	FACS	10× Genomics; SMART-Seq2	GSE103354
Structural cells	2020 (52), Nathan D. Jackson	Human	Human bronchialtracheal, nasal airway epithelial	Airway epithelial cells, ciliated cells, mucus secretory cells	IL-13	IL-13 induces emergent mucus secretory expression states for airway epithelial cells	Dissociation solution	ICELL8 platform, Illumina HiSeq 2500	GSE145013
Structural cells	2019 (53), Jamie L. Everman	Human	Lung, tracheal, nasal airway epithelial cells	Airway epithelial cell, alveolar type 1 and type 2 cells, lung immune cells, endothelial cells	–	CDHR3 was highly and exclusively expressed in ciliated cells	EpCAMMicroBeads	ICELL8, Illumina HiSeq 2500	–
Immune and structural cells	2019 (54), Felipe A. Vieira Braga	Human	Lung	Structural and inflammatory cells	*MUC5AC*, IL-4/IL-13 signaling	Structural cell communication decreases with increased Th2 cell interaction	FACS	10× Genomics; SMART-Seq2	GSE130148, EGAS00001002649
Immune and structural cells	2021 (55), Jeffrey N. Harding	C57BL/6J mice	Lung	Th17, epithelial cells, DCs	*Il-17, Egfr, Ahr/ARNT* nuclear translocation, tyrosine kinase c-src, *Erk1/2*	High levels of combustion- increased Il-17 signaling, *Ahr* activation, *Egfr* signaling, and T cell receptor and co-stimulatory signaling pathways in epithelial cells in asthma	Gentle MACS Dissociator	Drop-seq, Illumina Nextseq500	PRJNA666321

### Studies on immune cells associated with asthma

T cell subsets, as the key participants of airway inflammation in asthma, are the focus of research on the pathogenesis of asthma. There is meaningful heterogeneity between and within Th subsets. Th1/Th2 imbalance caused by T cell differentiation disorder is one of the important mechanisms of asthma (56, 57). In recent years, some new T cell subtypes and their roles in asthma have been gradually recognized, including regulatory T (Treg), Th17 etc (58).

Th cells play an important role in the pathogenesis of asthma, but their transcriptional characteristics in the pathological progression of asthma remain unclear. Christopher Andrew Tibbitt performed scRNA-seq on Th cells in the house dust mite (HDM) model (49), and found that there were a large number of Foxp3^+^Treg, Th1, Th2 and Th17 cell subsets in the airway, while there were fewer Th-related cell subsets in lung tissue and lymph nodes. In this study, SMART-Seq2 platform was used to perform scRNA-seq on CD3^+^CD4^+^CD44^+^ Th cells in the airway. Through two independent experiments, the expression of 2000-2500 genes was detected in each cell, and a total of more than 12000 genes were detected in all cells, of which 1971 genes were significantly differentially expressed. Further analysis showed that these CD3^+^CD4^+^CD44^+^Th cells were divided into 6 subpopulations, all expressing *Cd4, Cd3e, Cd3g, Cd3d* and *Cd44. Cd200r1, Il6, Plac8* and *Igfbp7* were characteristic genes of Th2 cells, which were significantly enriched in the airway. ScRNA-seq revealed the diversity of Th cells, enriched the related pathways of lipid metabolism in Th2 subsets, elucidated the pathways of Th2 cell differentiation, and suggested that glycolysis and lipid metabolism play a significant role in driving Th2 cell-mediated immune response (49).

Grégory Seumois et al. analyzed the scRNA-seq of Th cells and Treg cells from asthmatics with HDM allergy and revealed new T cell subsets (36). Th and Treg subsets expressed interferon response signature in asthmatic patients without HDM allergy compared with asthmatic patients with HDM allergy. *TNFSF10* was enriched in these subsets, and its product, tumor necrosis factor-related apoptosis-inducing ligand, inhibited Th cell activation (36). These findings suggested that these subsets might suppress allergic responses, which helped to explain why only a small percentage of people developed Th2 responses to the nearly ubiquitous allergens. In peripheral blood of patients with allergy and asthma, allergen-specific CD4^+^ T cells secreted IL-9, which was significantly reduced after successful immunotherapy, suggesting that IL-9 was an important secreted product of long-term stimulation of allergenic T cells (36, 59).

Similarly, Benjamin J. Ulrich et al. found that IL-9, which was produced by allergen-specific recall and amplified by IL-33, was required for recall responses (45). More importantly, they discovered a special subset of IL-9-secreting CD4^+^ ST2^+^ T cells by scRNA-seq and scATAC-seq, which were tissue resident cells and acted as a long-term memory population, mediating rapid responses to allergen challenges. Meanwhile, using whole lung scRNA-seq, they found that IL-9 could affect the expansion and gene expression of many cells, such as CD4^+^ T cells, ILC2s and B cells. ScRNA-seq analysis identified and discovered the roles of IL-9-secreting CD4^+^ ST2^+^ T subsets in specific environments and interactions with other cells, more specific and detailed than previously known T cell subsets.

As antigen-presenting cells, DCs play an important role in the allergen-driven Th2 immune response in asthmatic airways (60). Gentaro Izumi et al. identified five distinct lung conventional CD11b^+^ DCs (cDC2) clusters by scRNA-Seq technology, in which the Ly-6C^+^ cDC2 subgroup rapidly accumulated in the lung after allergic sensitization and could strongly promote Th17 differentiation *in vitro*. Pseudo-temporal analysis of scRNA-Seq data and transfer experiments of cDC2 subsets revealed that partially mature Ly-6C^+^Ly-6A/E-CD301b-cDC2 expressed *Il1b* can promoted Th17 differentiation. In contrast, CD200^+^ mature cDC2 strongly induced Th2 differentiation, but not Th17 differentiation. Therefore, lung cDC2 promoted Th17 and Th2 differentiation at different maturation stages (48).

Although ILCs represent only a small part of the lung immune system, they play a crucial role in early response to pathogens and in facilitating the acquisition of adaptive immunity, and are active participants in the development of asthma (61). Antonia Wallrapp et al. used scRNA-seq to analyze ILCs in mouse lungs at steady state or stimulation with IL-25 and IL-33, and identified that Neuromedin U (NMU)-NMUR1 signaling promoted inflammatory ILC2 responses. Consistent with reports that NMU knockout mice had attenuated airway inflammation after sensitization (62), Antonia Wallrapp et al. found that NMU, NMUR1, could activate ILC2 *in vitro*, and concurrent administration of NMU and IL-25 amplified inflammation *in vivo*. Conversely, loss of this signal reduced the number and function of ILC2s and altered the transcriptional program after excitation (40).

Mukesh Verma et al. suggested that in asthma, ILCs and other cells establish a mutually balanced gene repression program (repressors *Nr4a2, Zeb1, Bach2 and JunD*) and preparation programs (signaling activators *Fhl2, FosB, Stat6, Srebf2 and MPP7*) in response to repeated stimulation of allergens. ILC2 were found to play an important role in memory-driven asthma. Reciprocal regulation between these two programs established and maintained memory-driven asthma. Activation of *Fhl2* by recall allergen stimuli downregulated *Bach2* and *JunD* and activated the ERK1/2-AP1 and STAT6 pathways, inducing a memory-driven asthma phenotype (42).

Lingli Wang et al. (43) established a mouse model of HDM/LPS-induced exacerbation of steroid-resistant asthma and analyzed immune cells in the lung by scRNA-seq. CD11b^+^macrophages, multiple DCs subpopulations, and ILC2 were found to be potentially involved in steroid-resistant AHR and airway inflammation. They identified that IL-13 was predominantly expressed by CD8^+^ memory T cells, ILC2, and basophils after HDM and LPS challenge, and its expression was largely steroid-resistant in the first two cells, suggesting that IL-13 was a key cytokine driving the pathogenesis of asthma exacerbation. At the same time, they identified multicellular signaling pathways (“EIF2 signaling”, “oxidative phosphorylation”, “Rho family GTPases signaling”) that were strongly associated with disease progression during exacerbation. Their study found that common or unique host responses at the single-cell level were regulated by a large number of low-risk intracellular events that were unlikely to be easily identified by conventional analytical methods, showing the research advantages of scRNA-seq. Furthermore, their single-cell analysis of lung immune cells added to the understanding of the cellular composition of lung and provided an important resource of information on immune cell networks in the lung during asthma exacerbation and steroid resistance, providing a comprehensive reference data of mouse cell atlas.

Neutrophils are crucial in type 2 allergic immunity, as well as the pro-allergic environment (low-dose of microorganisms, respiratory viral infections, and air pollutants) and host allergy susceptibility. Using scRNA-seq technology to study three different mouse models of allergic asthma, Coraline Radermecker et al. found that neutrophil extracellular traps released CXCR4^hi^ lung neutrophils as an early stage of type 2 inflammation triggering factors. These neutrophils promoted allergen uptake by lung CD11b^+^Ly-6C^+^ DCs and increased susceptibility. In this study, scRNA-seq analysis confirmed that there were significant differences in lung neutrophils in the inflammatory environment induced by low-dose and high-dose lipopolysaccharide exposure (41). Furthermore, Zhiwei Li et al. identified the inflammatory neutrophils with specific molecular features of innate immune memory (G-CSFR^hi^FcγRIIb^+^) by scRNA-seq on lung tissues of asthmatic mice, which suggested that inflammatory memory neutrophils might aggravate asthma (46).

Ailu Chen et al. used DropSeq for scRNA-Seq technology to study the response of peripheral blood mononuclear cells (PBMCs) from severe asthmatic individuals and healthy individuals to the TLR3 agonist Poly I:C. They found that CD4^+^ T cells were predominant cell type in severe asthma, and exhibited a pro-inflammatory signature of increased JAK1 expression. Following Poly I:C stimulation, PBMCs from severe asthma strongly induced the interferon pathway compared with controls. This study found that the response of cells from asthmatics to interferon stimulation was not impaired (37).

Kaveh Moghbeli et al. measured the cell specificity of the bronchoalveolar lavage (BAL) cAMP gene network through transcriptome analysis of human lung parenchyma, BAL, and monocyte cell lines. They found that expression of the BALcAMP gene network was restrained under the influence of severe asthma and prolonged β-agonist exposure. Disturbances in this signaling were seen in many cell populations, especially macrophages in the alveolar and interstitial spaces, and might have implications for immune effector function (44).

Hui Li et al. used scRNA-seq to explore the immune status of BALF in patients with asthma exacerbations and found increased levels of CD8^+^ T cells, several monocyte clusters, and a monocyte-derived macrophage subset (39).They also found that an additional group of core exacerbation-associated modules was activated, including eukaryotic initiation factor 2 signaling, ephrin receptor signaling, and C-X-C chemokine receptor type 4 signaling in the subpopulations of CD8^+^ T cells (C1-a) and monocyte clusters (C7 clusters), which were associated with infection. Their analysis provided an insightful framework for understanding the shared and distinct expression patterns of inflammatory genes at the individual-cell level.

Airway microbial disturbances are strongly linked to the onset and severity of asthma, but the underlying link is unknown. Daniel Spakowicz et al. developed an integrated dimensionality reduction and statistical modeling approach, linking human genes, cells as well as microbes and identified their relationships. They validated the method with scRNA-seq and studied sputum from asthmatics and found unknown interactions between microbes and genes, such as mast cells responding to *Haemophilus* and *Pasteurella via* IL1B, while eosinophils responding to *Candida via* GCSAML (38).

### Studies on structural cells associated with asthma

Mucin is the main component of respiratory mucus, which can moisten airway, absorb impurities, clean and expel foreign bodies. If the secretion of mucin increased in the airway, small airway lumen will be obstructed or spasm, which will induce asthma. Therefore, epithelial disfunction and airway remodeling (pathological mucus increase due to goblet cell metaplasia, submucosal gland hyperplasia, etc.) are typical features of asthma (20). The development of scRNA-seq technology has enabled people to clearly understand the role of various structural cells in the airway during asthma attacks.

The type 2 inflammatory factor IL-13 has vital roles in structural cells (22, 23), such as epithelial goblet cell metaplasia (24) and mucin glycoprotein MUC5AC secretion (25, 26). Nathan D. Jackson et al. performed scRNA-seq using cultured airway epithelial cells and found that IL-13 induced an emergent mucus secretion state in multiple cell types. It was demonstrated that IL-13-induced airway mucus obstruction was an important feature of type 2 high asthma, and that IL-13 generally reduced innate airway defenses, resulting in a pathological mucus secretome that prevented mucociliary motility (52). Asthma is associated with changes in the structure and function of the airway epithelium, a key site of SARS-CoV-2 infection (63, 64). The altered expression of many asthmatic airway epithelial genes is driven by the type 2 cytokine IL-13 (26). Luke R. Bonser et al. used scRNA-seq to identify cytokine-induced changes in SARS-CoV-2-related gene expression in human bronchial epithelial cells (HBECs) and correlated with gene expression changes in the airway epithelium of patients with mild-to-moderate asthma (65). Their results identified that many IL-13-regulated SARS-CoV-2-related genes detected in HBECs were also altered in type 2 high asthma. IL-13 pretreatment reduced viral RNA recovery from SARS-CoV-2 infected cells and reduced dsRNA, a marker of viral replication, and mucus also inhibited viral infection. It was suggested that the stimulation of HBECs with IL-13 affected the expression of many SARS-CoV-2-related genes and largely inhibited the infection of these cells against SARS-CoV-2, which just explained the relatively low prevalence of asthma in patients diagnosed with COVID-19.

MiR-141 is abundantly expressed in branching airways (66) and has many predicted mucus-related targets (67). Sana Siddiqui et al. found that disruption of miR-141 resulted in reduced numbers of goblet cells, MUC5AC and total secreted mucus induced by IL-13 stimulation. The results were consistent with a decrease in goblet cell gene expression and an increase in basal cell gene expression obtained by scRNA-seq analysis (50).

Jamie L. Everman et al. performed scRNA-seq on whole lung cell suspensions and found that the cadherin-related family member 3 (CDHR3) protein was exclusively expressed in ciliated airway epithelial cells. They also revealed the underlying mechanism on the risk of asthma exacerbations conferred by the CDHR3 rs6967330 variant (53).

Using scRNA-seq analysis, Jeffrey N. Harding et al. found increased *Il-17* signaling, *Ahr* activation, *Egfr* signaling, T cell receptor and co-stimulatory signaling pathways in epithelial cells when exposed to particulate matter. Their data proved that particulate matter promoted an eTh17-specific inflammatory response leading to neutrophilic asthma through pathways in epithelial, DCs and T cells (55).

As a relatively rare cell type, lung ionocytes can express the ion transporter and cystic fibrosis *CFTR* gene and play a role in regulating the transport of ions and fluids through the airway epithelium and the pH of the mucosal surface. Daniel T. Montoro et al. studied the cellular composition of mouse tracheal epithelium and identified FoxI1-positive lung ionocytes using scRNA-seq and *in vivo* lineage tracing. Deletion of the related gene *Foxi1* in mouse ionocyte might result in loss of *Cftr* expression and disrupted airway mucus physiology (51).

The interaction of lung structural cells and immune cells is critical for maintaining lung homeostasis. Felipe A. Vieira Braga et al. used scRNA-seq to understand the cellular distribution of the lower airways in healthy and asthmatic patients. At the same time, they identified a new subset of tissue-resident memory T cells and a new mucociliary cell state in asthmatic airway epithelium. In addition, they found other asthma-related changes, including an increase in goblet cells, intraepithelial mast cells, and pathologic effector Th2 cells in the airway wall. Analysis of cell interactions in healthy subjects and asthmatic patients showed that structural cell interactions were significantly reduced with a significant increase in Th2 cells interactions. This study provided new insights into changes in epithelial cells, suggesting that altered patterns of communication between immune and structural cells underlied asthmatic airway inflammation (54). It’s worth noting that Vieira Braga et al. did not identify specific clusters of neuroendocrine cells by scRNA-seq, so supervised analysis using neuroendocrine cell marker genes identified a small number of cells with neuroendocrine-like features present only in lower airways.

## Discussion

The advent and rapid development of scRNA-seq technology has made it possible to study individual cells in tissues in depth, classify these cells into cell types, and characterize variations in their molecular profiles as a function of genetics, environment, cell–cell interactions, developmental processes, aging, or disease. Only by understanding the heterogeneity of cells can we make a breakthrough in the study of their common manifestations. The pathogenesis of asthma is affected by a variety of factors. There are acute phase and chronic remission phase of asthma, and the status of related cells may be different in different periods. The identification pathways and molecular events of asthma exacerbation are key areas of asthma. However, previous asthma transcriptomic studies have been limited in their understanding of the cellular and molecular composition of the lung immune response by the lack of adequate tools to study differences between cells that are histologically indistinguishable. Th cells, in particular, have subsets that are limited by assessment at independent time points and in response to different cytokine environments (68–70). These differences need to be further verified by methods such as single-cell sequencing.

The currently published literatures on lung tissue scRNA-seq has increased the understanding of lung cell composition and provided a comprehensive and important source of data for constructing cell atlas of human and mouse. Kyle J. Travaglini et al. applied droplet- and plate-based scRNA-seq to systematically identify the cell populations and gene expression profiles involved in lung tissue, created a molecular cell atlas (71). This atlas elucidated the biochemical functions of lung cell types and cell-selective transcription factors, while also provided a molecular basis for studying how lung cell identity, function, and interactions altered in development and asthma.

Although scRNA-seq technology has matured, there is still room for further improvement. Firstly, the preparation of tissue single cell suspensions is complicated, which poses a challenge to the classification of some cell populations in samples. Some single-cell isolation technology may have impact on cell integrity, resulting in data distortion or loss (29), such as FACS sorting may have non-negligible effects on cell viability of Chinese Hamster Ovary cells and for a human monocytic cell line (THP1) (72). The different types of cells that make up the tissue have different conditions of response to different digestive enzymes. In order to obtain as many kinds of tissue cells as possible, it is necessary to explore experimental methods of enzymatic digestion. Secondly, maintaining fidelity and avoiding bias during large-scale amplification is not an easy task. Moreover, another limitation of this technology is the relatively shallow sequencing “depth” of individual cells, which largely limits the discovery of high expressed genes (73), such as neuroendocrine cells. Limits on the number of cells that can be sequenced can hamper the identification of rare cells. The cost of scRNA-seq technology is still high, which affects its wide application. The bioinformatics analysis of scRNA-seq is currently carried out by experts in professional fields. Creating simplified data analysis methods and unified processing strategies is also an urgent problem to be solved.

Currently, there are only a limited number of scRNA-seq studies on asthma, and most have used mice models. Although there are many similarities between asthma patients and airway allergic mice, and mice are widely used in preclinical studies of asthma, there are crucial differences between human and mice lungs at the anatomical and cellular levels that should be taken with caution when extrapolating human asthma from mice data (74, 75). Lung organoids also have considerable potential to discover new therapeutic approaches for diseases such as asthma (76), and scRNA-seq can be used to identify the origin of cellular composition of lung organoids (77) to explore their differentiation mechanisms and pathways involved (78). Recent studies showed that single-cell omics researches had been extended from transcriptome to multi-omics, and single-cell multi-omics sequencing technology will more comprehensively and accurately reveal the specific mechanism of various lung cells and their subtypes in the pathogenesis of asthma.

## Author contributions

WW, WT and JD carried out the concepts, design, definition of intellectual content, literature search and manuscript preparation. WW, WT drafted the manuscript and figures. ML carried out the literature search and manuscript modification. JD helped perform the manuscript with constructive discussions. FT and JC provided assistance for literature search and manuscript editing. All authors have read and approved the content of the manuscript.

## Funding

This work was supported by grants from the National Natural Science Foundation of China (Grant No. 82174170), Research Fund of Huashan Hospital affiliated to Fudan University (2021QD042), the Innovative Research Team of High-level Local Universities in Shanghai, and Expert workstation for Jingcheng Dong in Yunnan Province (20210101) Province (20210101).

## Conflict of interest

The authors declare that the research was conducted in the absence of any commercial or financial relationships that could be construed as a potential conflict of interest.

## Publisher’s note

All claims expressed in this article are solely those of the authors and do not necessarily represent those of their affiliated organizations, or those of the publisher, the editors and the reviewers. Any product that may be evaluated in this article, or claim that may be made by its manufacturer, is not guaranteed or endorsed by the publisher.
